# Human In-Vivo Bioassay for the Tissue-Specific Measurement of Nociceptive and Inflammatory Mediators

**DOI:** 10.3791/1074

**Published:** 2008-12-01

**Authors:** Martin S Angst, Martha Tingle, Martin Schmelz, Brendan Carvalho, David C Yeomans

**Affiliations:** Department of Anesthesia, Stanford University School of Medicine; Department of Anaesthesiology, University of Heidelberg

## Abstract

This in-vivo human bioassay can be used to study human volunteers and patients. Samples are collected from pertinent tissue sites such as the skin via aseptically inserted microdialysis catheters (Dermal Dialysis, Erlangen, Germany). Illustrated in this example is the collection of interstitial fluid from experimentally inflamed skin in human volunteers. Sample collection can be combined with other experimental tests. For example, the simultaneous assessment of locally released biochemicals and subjective sensitivity to painful stimuli in experimentally inflamed skin provides the critical biochemical-behavioral link to identify biomarkers of pain and inflammation. Presented assay in the living human organism allows for mechanistic insight into tissue-specific processes underlying pain and/or inflammation. The method is also well suited to examine the effectiveness of existing or novel interventions - such as new drug candidates - targeting the treatment of painful and/or inflammatory conditions. This article will provide a detailed description on the use of microdialysis techniques for collecting interstitial fluid from experimentally inflamed skin lesion of human study subjects. Interstitial fluid samples are typically processed with aid of multiplex bead array immunoassays allowing assaying up to 100 analytes in samples as small in volume as 50 microliters.

**Figure:**
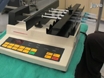


## Protocol

### Induction of experimental inflammation:

A first-degree sunburn is induced in non-tanned skin of a thigh with aid of a calibrated, ultraviolet B source (Saalmann Multitester SBB LT 499, Saalmann GmbH, Hereford, Germany). The sunburn is induced 24 hours before starting a microdialysis experiment by applying three times the amount of UVB light necessary to cause complete reddening of exposed skin, or three times the minimal erythemal dose (MED). The MED is determined in each subject before study initiation. Accurate delivery of energy is assured with a UV-meter (RM 12, Dr. Grobel UV-Electronic GmbH, Ettlingen, Germany).

### Catheter placement and sample collection:

Prepare a staging area with sterile gloves, 1 sterile needle holder, 1cc luer tip syringe with a 23 gauge (G) needle, 1cc syringe with a 30G needle, 1% lidocaine, alcohol swabs, 2x2 sterile gauze pads, microdialysis catheters attached at one end to a 4cm 23G needle (Dermal Dialysis, Erlangen, Germany), and 1.5 ml Microcent collection tubes (E&K Scientific, Santa Clara, CA.) with 4µl protease inhibitor solution per 100µl sample on dry ice. protease inhibitor solution is made per manufacturer’s instruction using protease inhibitor cocktail tablets (Roche Diagnostics, GmbH, Mannheim, Germany).
Prepare a 1% human albumin solution in lactated ringer’s solution and draw it into the 1cc luer tip syringe, making sure there are NO air bubbles remaining in the syringe.
Load the syringe with the 1% human albumin-enriched ringer’s lactate solution into the cradle (microdialysis multi-syringe rack, Harvard Apparatus, South Natick, MA) mounted onto a high precision infusion pump (Pump 22, Harvard Apparatus, South Natick, MA). Make certain that the bar of the infusion pump driving the syringe is in full contact with the plunger of the syringe. Set the pump rate after confirming that the syringe diameter has been accurately entered into the pump. Commonly used infusion rates range between 2 to 4µl/min.
Define the area you plan to collect your sample from. When sampling from an artificially created inflamed area, such as the sunburn described above, plan on inserting the catheter at the edge of the sunburn.
Clean a small area at opposite edges of the lesion, or site you intend to collect your sample from, with alcohol. These cleaned areas will be the intended insertion and exit points for the catheter. Inject just enough lidocaine intra-cutaneously (evidenced by blanching of the skin on injection) at these sites to create a very small bleb (maximum of 2mm). Injection of such limited amount of intra-cutaneous lidocaine at the entry and exit points of the microdialysis catheter should not alter the results of the bioassay but does make the insertion of the catheter significantly less painful for study subjects.
Using aseptic techniques, insert the microdialysis catheters with aid of attached 23G needle over the length of the explored skin area. The depth of insertion is at the interface between cutis and subcutis. Insertion at an adequate depth can be verified optically. As the needle and the attached catheter are moved forward the needle should cause a little up-folding of the skin as well as some blanching of the skin. No bleeding should occur. If bleeding does occur this indicates that the needle is located deeper than intended and the depth of insertion has to be adjusted. Once the needle has been pushed through the exit point of the skin, flush the catheters with 1% albumin enriched ringer’s lactate solution until you see droplets along the length of the catheter. This is done by carefully inserting the needle end of a syringe (23G needle) into the distal end of the microdialysis catheter, and slowly injecting solution.
After flushing the catheter pull gently on the needle to feed the attached catheter through the skin. Gentle handling is required because the catheters are small-diameter, and consequently fragile. Pull the catheter until it is completely threaded through the skin, i.e., the base of the larger plastic tubing embedding the distal end of the catheter touches the skin at the entry point. As mentioned above, little to no bleeding at the end of the procedure confirms optimal catheter placement.
Attach the distal end of the catheter to the tip of the 23G needle of the syringe resting in the rack of the infusion pump. Care is required to avoid puncturing the needle through the wall of the catheter, which would cause potential leakage.
Cut the needle off the proximal end of the catheter.
Using ¼-inch pink tape, attach the prepared 1.5ml Microcent collection tube at the skin close to the exit site of the catheter. Place the tip of the catheter into the collection tube to allow collection of microdialysate. Start the perfusion pump at predetermined rate and intermittently check that delivered and collected volume match up. In case the volumes do not match up, leakage from the skin entry site should be excluded (can sometimes be resolved by pulling the part of the catheter that is embedded by the plastic tubing more tightly onto the skin entry site). Alternatively, a catheter may need to be replaced if delivered and collected volumes do not match up.
Once sampling is complete, the samples are placed on dry ice until storage at -80ºC.

## Discussion

The combined use of microdialysis techniques and multiplex immunoassay technology is a valuable real-time, in-vivo human bioassay providing insight into biochemical events in tissue of interest. Combined with behavioral tests such as pain tests, the method allows studying the complex interactions of biochemicals mediating pain and inflammation. The method is promising for 1) identifying tissue specific biomarkers mediating pain and inflammation, 2) providing mechanistic insight into the pathology of inflammatory and chronic pain conditions with peripheral tissue pathology, and 3) exploring analgesic/anti-hyperalgesic and anti-inflammatory actions of novel drug candidates and interventional therapies.
